# Health equity implications of COVID-19 on dementia care in community for people of African descent

**DOI:** 10.1093/geroni/igab046.3605

**Published:** 2021-12-17

**Authors:** Mary Lukindo, Barbara Hamilton-Hinch, OmiSoore Dryden, Katie Aubrecht

**Affiliations:** 1 Dalhousie University, Dalhousie University, Nova Scotia, Canada; 2 St. Francis Xavier University, St. Francis Xavier University, Nova Scotia, Canada

## Abstract

This presentation shares results from an analysis of interviews conducted to understand the health equity implications of COVID-19 responses on dementia care in the community for people of African descent in Nova Scotia, Canada. Interviews were embedded within a larger multi-method rapid research project that aimed to assess the impact of COVID-19 on dementia care for geographically and socially marginalized groups in the province. Data from the interviews was analyzed using a constructivist thematic analysis method, guided by an intersectional theoretical scaffold. Three themes were identified related to systemic barriers, mental health, system navigation and self-care, and collected under the overarching theme of ‘facing the unknown with dementia’. Results emphasized the value and notable absence of community driven, culturally specific dementia programs, resources and navigators for people living with dementia, family caregivers and care workers of African descent. Participants identified lack of health system and care provider knowledge and understanding of the cultures and histories of people of African descent as a barrier to quality care and to addressing dementia-related stigma. Conversations focused on the need for practical and accessible tools, strategies and perspectives responsive to the actually lived realities and needs of people in community, and for research that actually contributes to individual and collective life in tangible, timely and culturally meaningful ways. Recommendations focus on the importance of centering community in dementia care programs, policy, practice and research to improve services and supports for people of African descent.

